# Jasmonic Acid-Dependent Defenses Play a Key Role in Defending Tomato Against *Bemisia tabaci* Nymphs, but Not Adults

**DOI:** 10.3389/fpls.2018.01065

**Published:** 2018-07-20

**Authors:** Peng-Jun Zhang, Yu-Chen He, Chan Zhao, Zi-Hong Ye, Xiao-Ping Yu

**Affiliations:** Zhejiang Provincial Key Laboratory of Biometrology and Inspection and Quarantine, College of Life Sciences, China Jiliang University, Hangzhou, China

**Keywords:** *Bemisia tabaci*, jasmonic acid, salicylic acid, plant defense, tomato

## Abstract

The silverleaf whitefly *Bemisia tabaci* is an important and invasive crop pest in many countries. Previous laboratory studies with *Arabidopsis* demonstrated that *B. tabaci* can suppress jasmonic acid (JA) defenses and thereby enhance *B. tabaci* performance. Whether *B. tabaci* can suppress JA-regulated host plant defenses in field is unknown. In the present study, we found that, relative to wild-type (WT) tomato plants, transgenic tomato mutants that activated JA defenses (*35s::prosys*) or impaired JA defenses (*spr-2* and *def-1*) did not affect the survival or reproduction of *B. tabaci* adults in growth chamber experiments. In contrast, tomato mutants that activated JA defenses slowed *B. tabaci* nymphal development, while mutants that impaired JA defenses accelerated nymphal development. These effects of JA defenses on nymphal development were also documented under semi-field conditions. Changes in the expression of defense genes and in the production of phytohormones indicated that *B. tabaci* adults can suppress JA-dependent defenses after infestation for >72 h. The suppression of JA was correlated with the induction of salicylic acid (SA) in *B. tabaci*-infested leaves under laboratory and under semi-field conditions. If SA signaling was blocked, JA accumulation increased in infested leaves and *B. tabaci* nymphal development was delayed. These results indicate that, although JA signaling helps in mediating tomato responses against *B. tabaci* nymphs, *B. tabaci* can inhibit JA biosynthesis and its action in an SA-dependent manner.

## Introduction

Plants have various defenses against herbivores, and some defenses are induced. Induced plant defenses include the production of antibiotic or antixenotic metabolites that directly influence herbivore performance and behavior (Bleeker et al., 2009; Luan et al., 2013), and the release of volatiles that indirectly recruit parasitoids or predators to infested plants and that thereby indirectly affect herbivores (Turlings et al., 1990; Kessler and Baldwin, 2001; Dicke et al., 2009). The jasmonic acid (JA) signaling pathway is especially important in mediating induced plant defenses against herbivores (Ament et al., 2004; Kessler et al., 2004; Thaler et al., 2012), although salicylic acid (SA) and ethylene (ET) signaling pathways are also important in some cases (Penninckx et al., 1998; Zhang et al., 2013b). In tomato, for example, JA regulates the expression of defense-related genes (Farmer et al., 1992) and the emission of herbivore-induced volatiles that may attract natural enemies (Ament et al., 2004). Accordingly, the blocking of JA synthesis or action can increase the susceptibility of tomato plants to herbivores (Bosch et al., 2014), and can interfere with the pest control by natural enemies (Thaler et al., 2002).

Upon attack, plants perceive elicitors or herbivore-associate molecular patterns, and thereby trigger the JA signaling pathway and anti-herbivore defense responses (Schmelz et al., 2009). Growing evidence indicates that insect herbivores can exploit their elicitors or effectors to circumvent plant defenses for their own benefit (Kant et al., 2015). The butterfly *Pieris brassicae*, for example, utilizes egg-derived elicitors to inhibit the expression of JA-regulated defense genes in *Arabidopsis* (Bruessow et al., 2010). The honeydew secreted by the pea aphid *Acyrthosiph pisum* can suppress JA accumulation in broad bean plants (Schwartzberg and Tumlinson, 2014). Larvae of the Colorado potato beetle, *Leptinotarsa decemlineata*, exploit bacteria in their oral secretions to suppress JA-dependent defenses in tomato and to thereby promote larval growth (Chung et al., 2013). The suppression of JA-dependent defenses in above cases is mainly dependent on a functional SA-signaling pathway. However, some herbivores can suppress JA responses through other SA-independent mechanisms. For example, two spider mite species, *Tetranychus urticae* and *T. evansi* can suppress the defenses downstream of both JA and SA simultaneously in tomato (Sarmento et al., 2011; Alba et al., 2015). The leafhopper *Macrosteles quadrilineatus* suppresses JA defenses via an effector derived from a vectored phytoplasma, resulting in increased performance of its larvae (Sugio et al., 2011).

The silverleaf whitefly *Bemisia tabaci* is an important and invasive pest of crops worldwide. *B. tabaci* is a genetically diverse group, and more than 20 biotypes have been named from population of this species complex (Perring, 2001). The major global invasion event associate with *B. tabaci* is the invasion by the individuals of biotype B (Middle East–Asia Minor 1) which in the past 20 years has spread rapidly around the world (De Barro et al., 2011). *B. tabaci* has four larval stages. Except first instar larvae, other stage larvae are immobile and settle at a suitable feeding site until adult emerge. In general, the developmental duration of *B. tabaci* from egg to adult ranges from 19 to 24 days, which depends on host plant species (Zang et al., 2006). Studies with *Arabidopsis* showed that feeding by *B. tabaci* adults or nymphs can down-regulate JA-regulated defenses (Van de Ven et al., 2000; Kempema et al., 2007; Zarate et al., 2007) and that such down-regulation may be mediated by SA cross-talk (Walling, 2008). With the suppression of JA defenses, *B. tabaci* nymphal development was significantly accelerated on *Arabidopsis* (Zarate et al., 2007; Zhang et al., 2013a). Because the whitefly–*Arabidopsis* interaction rarely occurs in the field, however, the ecological relevance of the interaction is unclear. Remarkably, recent works have demonstrated that *B. tabaci* infestation can suppress the JA-regulated volatile emissions in Lima bean and tomato (Zhang et al., 2009; Su et al., 2018), as well as the expression of JA-dependent genes (Su et al., 2015). However, it still remains unknown whether *B. tabaci* feeding can suppress JA-regulated host plant defenses in field. In the present study, we compared the performance of *B. tabaci* adults and nymphs on wild-type (WT) tomato plants, on mutant/transgenic plants that constitutively activate or impair JA signaling pathways, and on WT plants treated with exogenous JA or SA. We then measured the changes in endogenous JA and SA and transcript levels of JA- and SA-regulated defense genes in response to *B. tabaci* infestation of tomato plants. Finally, we examined the effects of JA and SA defenses on *B. tabaci* development and determined the effects of SA signaling on the suppression of JA defenses by *B. tabaci* in semi-field experiment with transgenic *NahG* plants. Our results demonstrate that, although JA defenses in tomato are crucial for defending against *B. tabaci* nymphs, *B. tabaci* can suppress the JA defenses in an SA-dependent manner.

## Materials and Methods

### Plants and Insects

Wild-type tomato (*Solanum lycopersicum*) cv Moneymaker (MM) is the parental line for the SA-silenced *NahG*, which overexpresses the bacterial salicylate hydroxylase transgene (*NahG* gene). WT tomato cv Castlemart (CM) is the parental line for the JA-silenced *spr-2* and *def-1*, and also for *35s::prosys*, in which JA signaling is constitutive. The *spr-2* mutant carries a point mutation that results in loss of function of FATTY ACID DESATURASE 7 (FAD7), which is required for the JA biosynthesis (Li et al., 2003). The *def-1* mutant is deficient in the induction of both proteinase inhibitor I and proteinase inhibitor II following mechanical damage (Lightner et al., 1993). *35S::prosys* seeds were collected from a *35S::prosys* homozygote that had been backcrossed five times to its WT line cv CM (Howe and Ryan, 1999). Tomato plants were grown in 500 cm^3^ pots containing a commercial potting mix (Fafard Growing Mix 1, Agawam, MA, United States) and were kept in a climate-controlled chamber (25 ± 2°C, 60–70% RH, 10L: 14D photoperiod). Plants with four to five fully expanded leaves were used for experiments.

A colony of virus-free *B. tabaci* (Gennadius) MEAM1 (Hemiptera: Aleyrodidae) was maintained on WT MM plants in a separate climate-controlled chamber (25 ± 2°C, 50–60% RH, 10L: 14D). For all experiments, plants were infested with *B. tabaci* adults within 48 h after adult emergence.

Laboratory experiments 1–6 were performed in a climate-controlled chamber (25 ± 2°C, 60–70% RH, 10L: 14D photoperiod), and each tomato genotype or treatment was represented by 10–15 replicate plants, unless noted otherwise.

#### Performance of *B. tabaci* on Wild-Type (CM) and Mutant Tomato Plants

In laboratory experiment 1, 10 *B. tabaci* adults (1:1 male-to-female sex ratio) were released into a clip cage attached to the abaxial surface of a leaf (third or fourth leaf from the top) of WT CM, *spr-2, def-1*, and *35s::prosys* plants. The adults were allowed to feed and oviposit on the leaves. After 5 or 14 days, the fecundity was assessed by counting the eggs on the leaves with a binocular microscope.

In laboratory experiment 2, 20 *B. tabaci* adults were released into a clip cage (20.0 mm high, 45.0 mm diameter) attached to the abaxial surface of a leaf (third or fourth leaf from the top) of WT CM, *spr-2, def-1*, and *35s::prosys* plants. At 7, 10, and 14 days after the adults had been introduced, adult survival was consistently assessed by counting the surviving adults.

In laboratory experiment 3, 100 *B. tabaci* adults (a mixture of females and males) were released and allowed to feed on WT CM, *spr-2, def-1*, and *35s::prosys* plants in a ventilated cage (21.0 cm high, 13.5 cm diameter; one plant per cage). After 24 h of infestation and oviposition, the adults were removed from the plants by aspiration. At 21 days after introduction of adults, the number of nymphs and their developmental stages (first through fourth instars) were recorded. Developmental rate was estimated by calculating the proportion of nymphs represented by fourth instars (red-eye stage) on each plant.

### *B. tabaci* Development as Affected by Exogenous JA or SA

In laboratory experiment 4, JA or SA (Sigma-Aldrich) was dissolved in 0.2 mL of acetone and dispersed in water (containing 0.1% Tween 20) to produce a 0.5 mM JA or SA solution. Each WT CM plant was sprayed with 5.0 mL of the JA or SA solution with a hand-sprayer. Plants that were sprayed with 5 mL of water containing 0.2 mL of acetone and 0.1% Tween 20 were used as the control. Twenty-four hours later, the plants were placed in cages and infested with *B. tabaci* adults as described for laboratory experiment 3. After 24 h of infestation and oviposition, the adults were removed from the plants by aspiration. Twenty-one days after adults were placed on the plants, the proportion of nymphs represented by each instar was determined on each plant.

### Phytohormone and Gene-Expression Assay on Leaves Infested With *B. tabaci* Adults

In laboratory experiment 5, 100 *B. tabaci* adults were equally released into two clip cages that were secured to the abaxial surface of two leaves of one WT CM plant (about 1:1 male-to-female sex ratio). Plants clipped two cages without *B. tabaci* were used as the control. After 6, 12, 24, 48, 72, and 120 h postinfestation, leaves were collected from two infested or non-infested plants and were pooled as one biological sample respectively. These samples were frozen in liquid nitrogen and stored at -80°C until we extracted phytohormones and RNA. Each combination of time and treatment (with and without infestation) was represented by three biological replicates.

### Gene-Expression Assay on Leaves Infested with *B. tabaci* Nymphs

In laboratory experiment 6, 100 adult whiteflies (≤48 h after emergence) were collected and averagely released into two clip cages that secured to the abaxial surface of two leaves of one plant (50 adults/cage/leaf). Whiteflies were allowed to feed and oviposit on the leaves for 24 h. After that, all adult whiteflies were removed from the plants by aspiration. At 5, 7, and 10 days after the adults had been removed, leave that carried with nymphs were collected from two plants, and pooled as one biological sample.

### Quantification of Endogenous JA and SA

Endogenous JA and SA were extracted and quantified as described by Almeida Trapp et al. (2014). In brief, plant material (250–300 mg) was frozen and ground in liquid nitrogen. For quantification, [9, 10]-dihydro-JA (15 ng) and d6-SA (20 ng) were added as internal standards. Samples were analyzed using a GC/MS system (6890N/5973 MSD, Agilent Technologies, Inc., Palo Alto, CA, United States) equipped with an HP-5-MS column (30 m × 0.25 mm × 0.25 mm; J&W Scientific, Agilent Technologies). Endogenous JA, SA, and their internal standards were analyzed in full-scan mode.

### Total RNA Isolation and cDNA Synthesis

To minimize wounding- and dehydration-induced gene expression, leaf samples were quickly harvested and immediately frozen in liquid nitrogen. Each combination of time and treatment (with and without infestation) was represented by three biological replicates. Frozen samples were ground to a fine powder in liquid nitrogen with a pestle and mortar. Total RNA was extracted from 100 mg of each leaf sample using a plant RNA isolation kit (Axygen, Hangzhou, China) according to the manufacturer’s instructions. RNA concentration and purity were determined using a NanoDrop TM ND-2000 spectrophotometer (Thermo Scientific, Wilmington, DE, United States), and the integrity of RNA was also assessed by 1% agarose gel electrophoresis and ethidium bromide staining. First-strand cDNA was synthesized from 200 ng of RNA using a First-Strand cDNA Synthesis Kit (Bio-Rad, Hangzhou, China) according to the manufacturer’s instructions.

### Quantitative Real-Time PCR

The transcript levels of the *LoxD, PI-I, PI-II*, and *Pr-1b* were quantified by real-time quantitative RT-PCR (qRT-PCR). *LoxD* encodes a key enzyme in the octadecanoid pathway, which is important for JA biosynthesis in tomato (Heitz et al., 1997). Proteinase inhibitors I and II (*PI-I* and *PI-II*) are regulated by JA signaling and confer insect resistance in many *Solanaceous* plants, including tomato (Johnson et al., 1989; Li et al., 2002). *Pr-1b* is an SA-regulated pathogenesis-related (PR) gene in tomato (Jacobs et al., 2013). qRT-PCR was carried out on an ABI 7500 Real Time PCR System with a 96-well rotor. The amplification reactions were performed in a final volume of 20 μL that contained 10 μL of iQ^TM^ SYBR^®^ supermix (BioRad, Hangzhou, China), 0.8 μL of forward primer (5 μM) and reverse primer (5 μM) pairs, and 1 μL of cDNA first-strand template. Thermal cycling conditions were 5 min at 95°C, followed by 40 cycles of 15 s at 95°C, 15 s at 58–62°C, and 30 s at 72°C. Primers used for quantitative RT-PCR are listed in Supplementary Table S1. All reactions were run in duplicate, and average values were used in the analysis.

### Semi-Field Experiments

The effects of JA- or SA-dependent defenses on the performance of *B. tabaci* were assessed under semi-field conditions at China Jiliang University from June to September of 2016. In semi-field experiment 1, plants of each of the following six genotypes were planted in the field: WT MM, WT CM, *spr-2, def-1, 35s*, and *NahG*. Each genotype was represented by 30 plants, which were planted in five rows with 40 cm between adjacent plants. The plants of each genotype were grouped together, and adjacent genotypes were 2 m apart. Six weeks after planting, 15 plants were randomly selected from each genotype and were subjected to bioassay. Each plant was infested with 50 *B. tabaci* adults that were placed in a clip cage attached to the abaxial surface of one leaf. After 24 h, all adults were removed from the plants by aspiration. During the period of field experiments the average temperature was 31.5 ± 2.6°C, in which condition nymphal development will be significantly accelerated. Therefore, we observed the number of nymphs and their developmental stages (first through fourth instars) at 16 after introduction of adults, which is earlier than we observed in laboratory.

Semi-field experiment 2 was conducted to determine the effects of SA signaling on the suppression of JA defenses by *B. tabaci*. Thirty plants each of WT MM or SA-silenced *NahG* were planted in the field. Each of 12 plants per genotype was infested with 150 *B. tabaci* adults that were placed in three clip cages (50 adults/cage) attached to the third, fourth, and fifth leaf from the top. Twelve plants per genotype that had clip cages without *B. tabaci* were used as controls. At 3 and 5 days after infestation, leaves were collected from two randomly selected infested and non-infested plants per genotype and were pooled as one biological sample. At each timepoint, three biological samples were collected from *B. tabaci*-infested or uninfested plants. The samples were subjected to phytohormone analysis as described for laboratory experiment 5.

### Statistical Analysis

Data of female fecundity, adult survival, gene-expression, and phytohormone levels were analyzed by two-way ANOVA. Normalized gene expression was calculated using the 2^-ΔCt^ method with *GAPDH* as a reference gene, and values were subsequently log_2_ transformed for data analysis. The total number of eggs recorded after 5 or 14 days was divided by the number of females that were still alive at the end of the experiments. The percentage of fourth instars of *B. tabaci* on different plants were analyzed using the general linear model (GLM) for univariate analysis. If treatments were significant (*P* < 0.05), Tukey’s multiple-comparison tests were used to analyze significant differences between pairs.

## Results

### Adult Performance

**FIGURE 1:**
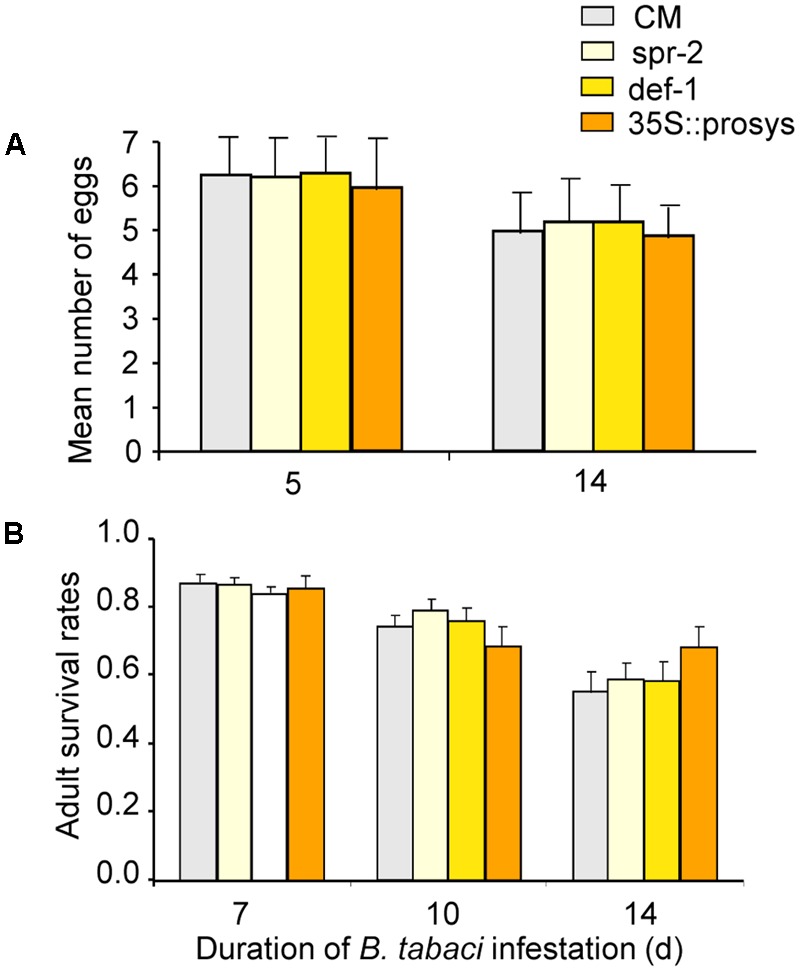
Performance of *B. tabaci* adults on wild-type (CM) and JA-signaling mutants of tomato. **(A)** Number of eggs laid per female per day on plants after 5 days of infestation. **(B)** The proportion of adults that had survived at 7, 10, and 14 days after introduction. Values are means (± SE) of 10–15 biological replicates.

The number of eggs laid per female per day did not differ on CM, *spr-2, def-1*, and *35s::prosys* plants (plant genotype: *P* = 0.95; time: *P* = 0.54; interaction: *P* = 0.91; **Figure 1A**). Adult survival rate did not differ on CM, *spr-2, def-1*, and *35s::prosys* plants (plant genotype: *P* = 0.13; time: *P* < 0.001; interaction: *P* = 0.89; **Figure 1B**). These data indicate that JA-dependent defenses do not affect the performance of *B. tabaci* adults.

### Nymph Performance

**FIGURE 2:**
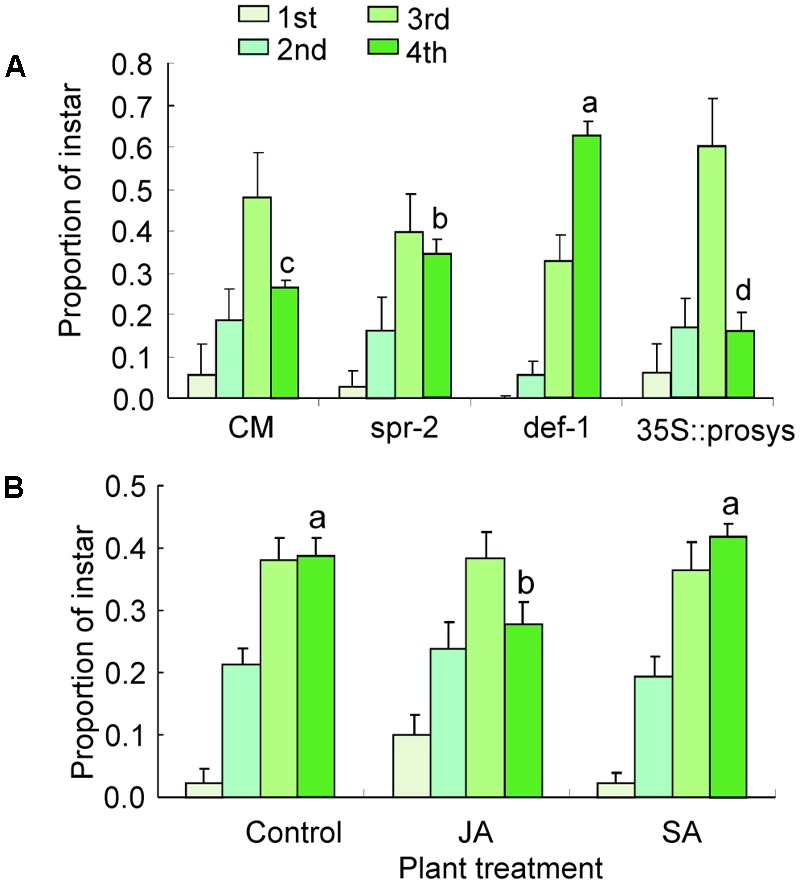
Performance of *B. tabaci* nymphs on tomato plants. **(A)** The proportion of nymphs represented by each instar at 21 days after adults were placed on CM and JA-signaling mutants of tomato; **(B)** The proportion of nymphs represented by each instar at 21 days after adults were placed on untreated (Control) and JA- or SA-treated CM plants. Values are means (±SE) of 10–15 biological replicates. Means for the fourth-instar with different letters are significantly different (univariate analysis with GLM, *P* < 0.05).

At 21 days postinfestation, the total number of nymphs survived did not differ on CM, *spr-2, def-1*, and *35s::prosys* plants (Supplementary Table S2). The proportion of fourth instars was significantly higher on *spr-2* and *def-1* plants than on CM plants (*def-1*: *P <* 0.001; *spr-2*: *P* < 0.001; **Figure 2A**). In contrast, the proportion of fourth instars was significantly lower on *35s::prosys* plants than on CM plants (*P <* 0.001; **Figure 2A**).

Likewise, exogenous JA or SA treatments have no effects on the total number of nymphs survived at 21 days postinfestation (Supplementary Table S2). When CM plants were sprayed with JA, the proportion of fourth instars was significantly reduced relative to the non-sprayed control (*P <* 0.001; **Figure 2B**). In contrast, the proportion of fourth instars was unaffected by exogenous SA treatment (**Figure 2B**). These data indicate that JA signaling pathway is important for defense against *B. tabaci* nymphs.

### Endogenous JA and SA Levels in Response to *B. tabaci* Feeding

**FIGURE 3:**
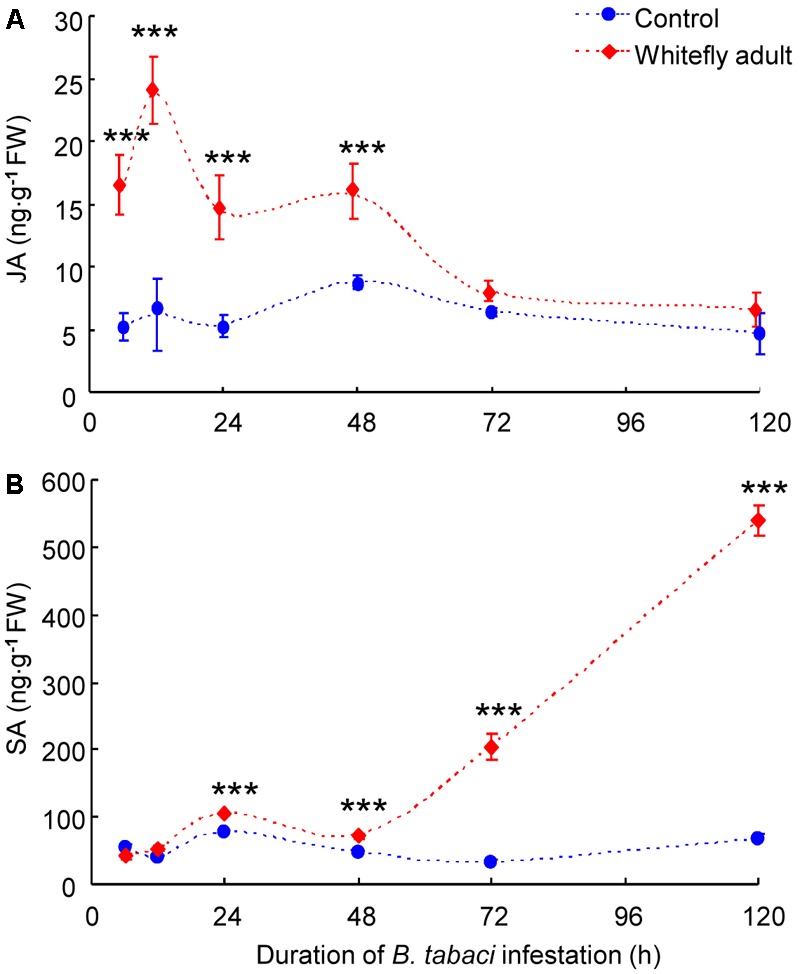
Levels of the phytohormones JA **(A)** and SA **(B)** in *B. tabaci*-infested and in non-infested leaves of WT CM plants. Values are means (±SE) of three biological replicates. Asterisks above bars indicate significant differences (two-way ANOVA; ^∗∗∗^*P* < 0.001). FW, fresh weight.

Whitefly infestation had a significant effect on JA accumulation (whitefly: *P* = 0.04; time: *P* = 0.24; interaction: *P* = 0.51). JA amount was significantly higher in *B. tabaci*-infested leaves than in non-infested leaves of CM plants at 6–48 h after infestation (*P* < 0.001) and peaked at 12 h (**Figure 3A**). After 48 h, JA amount in *B. tabaci*-infested leaves dropped and did not significantly differ from the amount in non-infested leaves (**Figure 3A**).

Whitefly infestation had a significant effect on SA accumulation (whitefly: *P* = 0.03; time: *P* = 0.43; interaction: *P* = 0.73). SA amount did not differ in *B. tabaci*-infested leaves vs. non-infested leaves of CM plants at 6 h or 12 h after infestation but was greater in the infested than in the non-infested leaves at 24–120 h after infestation (*P* < 0.001; **Figure 3B**).

### Defense-Related Gene Expression Induced by *B. tabaci* Adults and Nymphs

**FIGURE 4:**
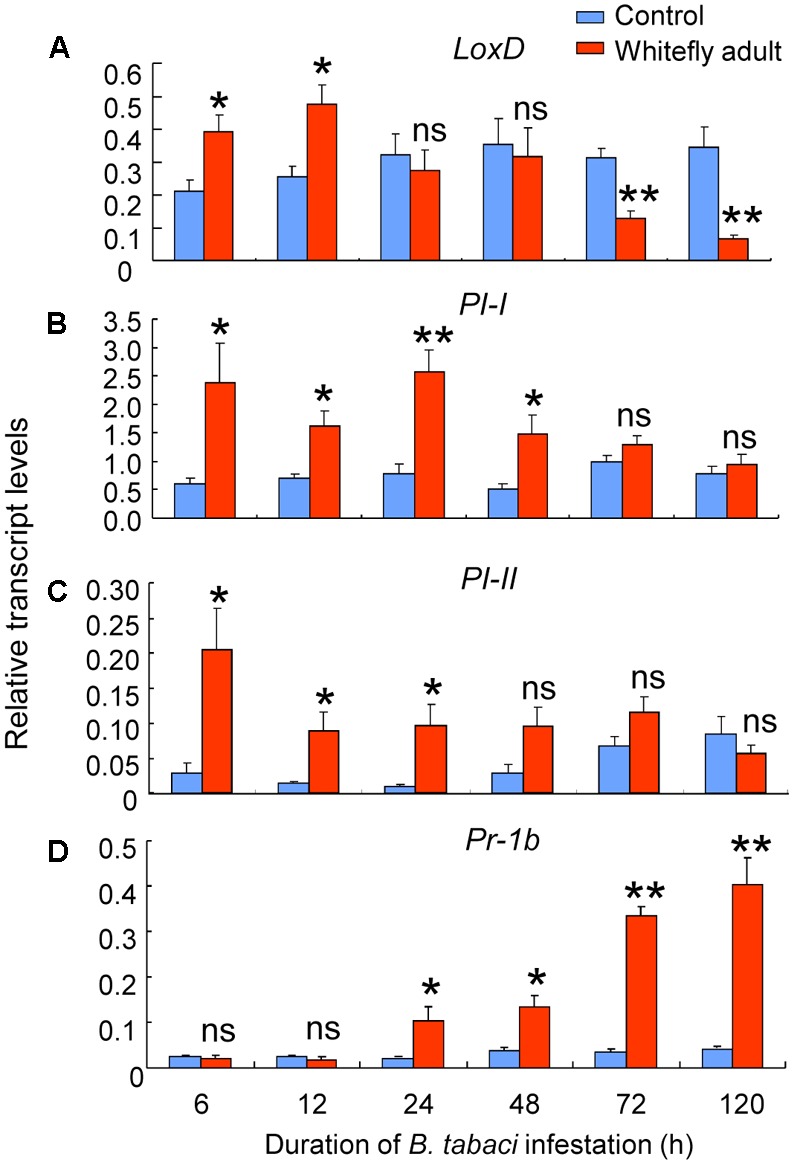
Expression of defense-related genes induced by *B. tabaci* adult infestation of wild-type CM plants. The transcript levels of *LoxD* **(A)**, *PI-I* **(B)**, *PI-II* **(C)**, and *Pr-1b* **(D)** were quantified by qRT-PCR and were normalized to the amount of *GAPDH* transcripts in each sample. Values are means (± SE) of three biological replicates. Asterisks above bars indicated significant differences compared to the non-infested control (two-way ANOVA; ^∗^*P* < 0.05, ^∗∗^*P* < 0.01). ns, not significant.

We next quantified the transcript levels of the following defense-related genes in infested and non-infested leaves of CM plants: *LoxD, PI-I, PI-II*, and *Pr-1b*. Whitefly adult infestation had significant effects on the expression of *LoxD* (whitefly: *P* = 0.31; time: *P* = 0.03; interaction: *P* < 0.001), *PI-I* (whitefly: *P* < 0.001; time: *P* = 0.06; interaction: *P* = 0.02), *PI-II* (whitefly: *P* < 0.001; time: *P* = 0.07; interaction: *P* = 0.03), and *Pr-1b* (whitefly: *P* = 0.01; time: *P* = 0.09; interaction: *P* = 0.01). For *LoxD*, transcript levels were increased by infestation at 6 and 12 h (6 h: *P* = 0.047; 12 h: *P* = 0.045), were unaffected by infestation at 24 and 48 h, and were reduced by infestation at 72 and 120 h (72 h: *P* = 0.008; 120 h: *P* = 0.01; **Figure 4A**). Infestation increased *PI-I* transcript levels at 6–48 h (*P* = 0.002–0.04; **Figure 4B**) and increased *PI-II* transcript levels at 6–24 h (*P* = 0.022–0.05; **Figure 4C**); thereafter, *PI-I* and *PI-II* transcript levels were similar in *B. tabaci*-infested and in non-infested leaves (**Figures 4B,C**). For *Pr-1b*, transcript levels were unaffected by infestation at 6 and 12 h but were increased at longer infestation times (24–120 h: *P* = 0.003–0.042; **Figure 4D**).

**FIGURE 5:**
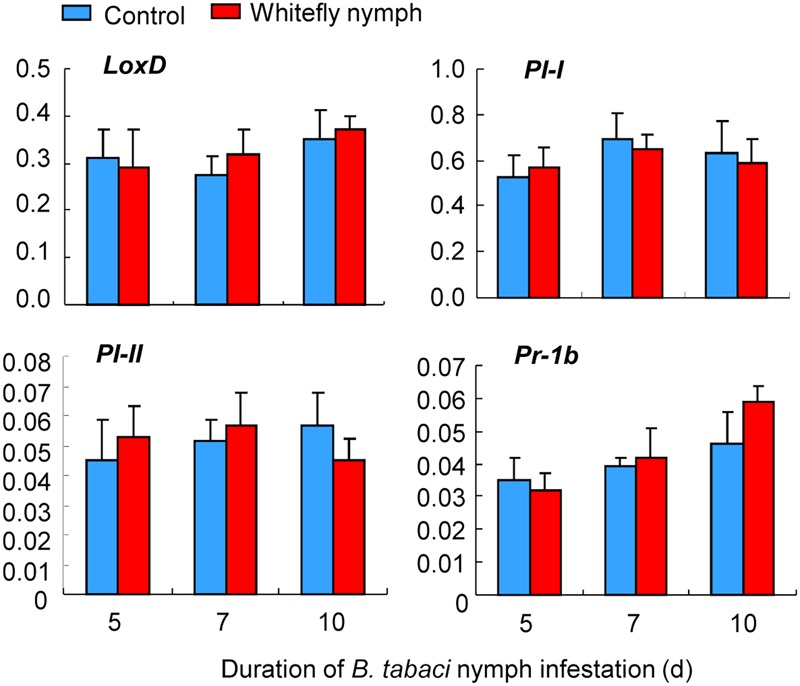
Expression of defense-related genes induced by *B. tabaci* nymph infestation of wild-type CM plants. The transcript levels of *LoxD, PI-I, PI-II*, and *Pr-1b* were quantified by qRT-PCR and were normalized to the amount of *GAPDH* transcripts in each sample. Values are means (±SE) of three biological replicates.

In contrast, whitefly nymph feeding did not affect the expression of *LoxD* (whitefly: *P* = 0.31; time: *P* = 0.23; interaction: *P* = 0.84), *PI-I* (whitefly: *P* = 0.38; time: *P* = 0.19; interaction: *P* = 0.73), *PI-II* (whitefly: *P* = 0.22; time: *P* = 0.14; interaction: *P* = 0.94), and *Pr-1b* (whitefly: *P* = 0.11; time: *P* = 0.33; interaction: *P* = 0.48; **Figure 5**).

### Nymph Performance Under Semi-Field Conditions

**FIGURE 6:**
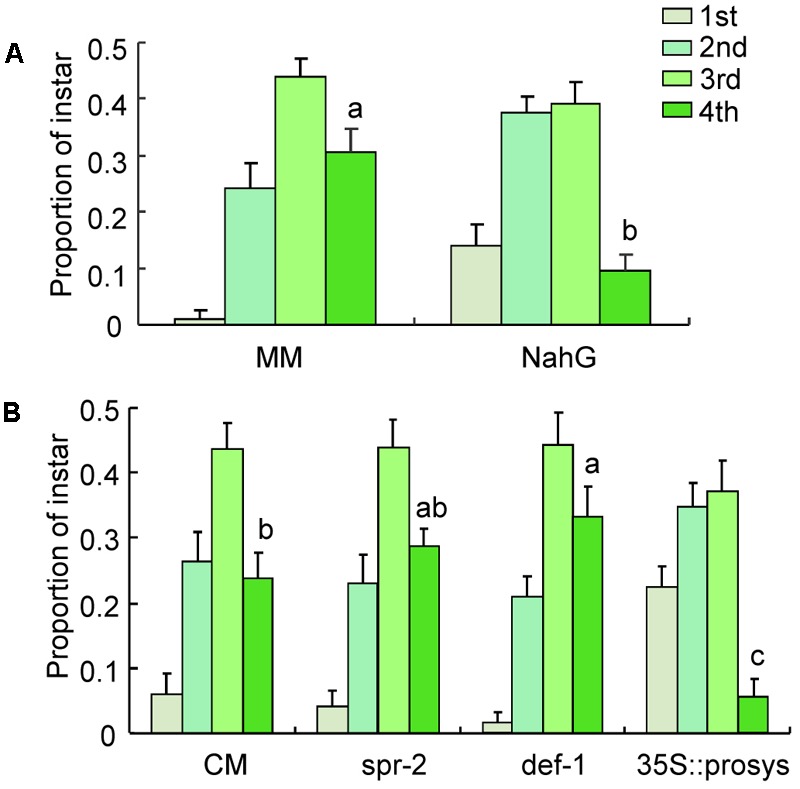
Performance of *B. tabaci* nymphs on different tomato genotypes under semi-field conditions. **(A)** The proportions of nymphs represented by each instar at 16 days after adults were placed on wild-type (MM) tomato plants and SA-silenced mutant (*NahG*) tomato plants; **(B)** The proportion of nymphs represented by each instar at 16 days after adults were placed on wild-type (CM) tomato plants and JA-signaling mutant tomato plants. Values are means (±SE) of 10 biological replicates. Means for the fourth instar with different letters are significantly different (univariate analysis with GLM, *P* < 0.05).

At 16 days postinfestation, the total number of nymphs survived did not differ on MM and *NahG* plants (Supplementary Table S3). The proportion of fourth instars was significantly lower on *NahG* plants, in which SA is silenced, than on MM plants (*P <* 0.001; **Figure 6A**). These results indicate that impairment of SA pathway slows *B. tabaci* nymphal development under semi-field conditions.

At 16 days postinfestation, the total number of nymphs survived did not differ on CM, *spr-2, def-1*, and *35s::prosys* plants (Supplementary Table S3). The proportion of fourth instars on *def-1* plants but not on *spr-2* plants was significantly higher than the proportion on CM plants (*def-1*: *P <* 0.001; *spr-2*: *P* = 0.69; **Figure 6B**). In contrast, the proportion of fourth instars was significantly lower on *35s::prosys* plants than on CM plants (*P <* 0.001; **Figure 6B**). These results indicate that JA signaling pathway is also important in defending against *B. tabaci* nymphs under semi-field conditions.

### Endogenous JA and SA Levels Induced by *B. tabaci* Under Semi-Field Conditions

**FIGURE 7:**
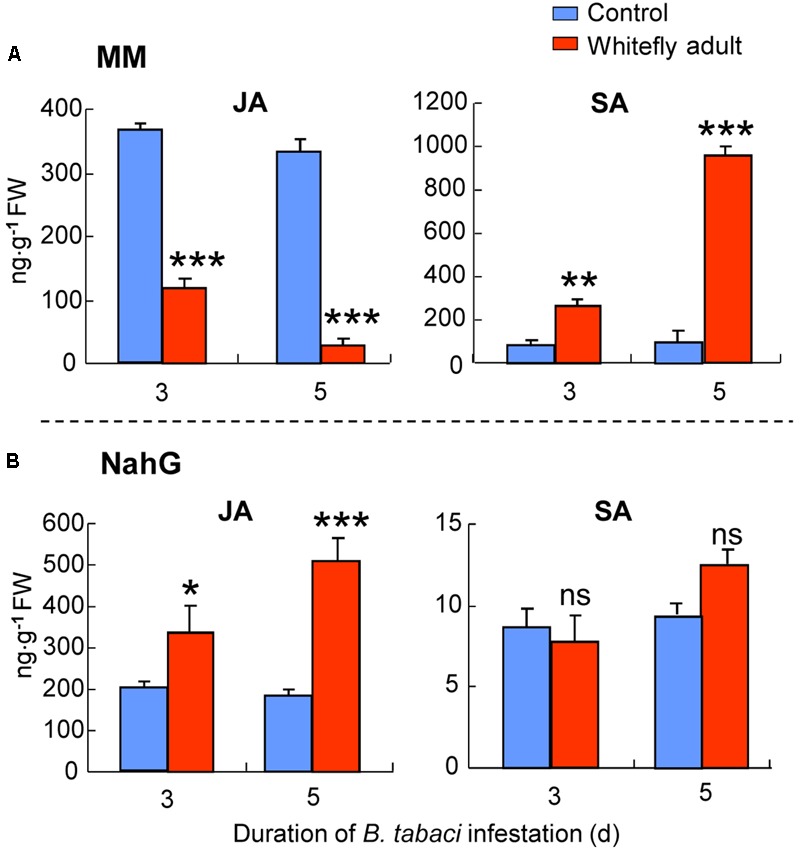
JA and SA levels in MM **(A)** or *NahG* plants **(B)** as affected by *B. tabaci* infestation under semi-field conditions. Values are means (±SE) of three biological replicates. Asterisks above bars indicated significant differences compared to the non-infested control (two-way ANOVA; ^∗^*P* < 0.05; ^∗∗^*P* < 0.01; ^∗∗∗^*P* < 0.001). ns, not significant. FW means fresh weight.

For MM plants in semi-field experiment 1, whitefly infestation had significant effects on JA (whitefly: *P <* 0.001; time: *P* = 0.23; interaction: *P* = 0.73) and SA (whitefly: *P <* 0.001; time: *P* = 0.48; interaction: *P* = 0.53) accumulation. The JA amount at 3 and 5 days was significantly lower in *B. tabaci*-infested plants than in non-infested plants (at 3 days: *P <* 0.001; at 5 days: *P <* 0.001; **Figure 7A**). In contrast, the SA amount of MM plants at 3 and 5 days was significantly higher in *B. tabaci*-infested plants than in non-infested plants (at 3 days: *P <* 0.01; at 5 days: *P <* 0.001; **Figure 7A**).

For *NahG* plants in semi-field experiment 1, whitefly infestation had significant effect on JA (whitefly: *P* = 0.003; time: *P* = 0.15; interaction: *P* = 0.40), but not SA (whitefly: *P* = 0.18; time: *P* = 0.26; interaction: *P* = 0.83) accumulation. The JA amount at 3 and 5 days was significantly higher in *B. tabaci*-infested plants than in non-infested plants (at 3 days: *P* = 0.04; at 5 days: *P <* 0.001; **Figure 7B**). In contrast, the SA amount of *NahG* plants was not significantly affected by *B. tabaci* infestation (**Figure 7B**).

## Discussion

Our data show that the reproduction and survival of *B. tabaci* adults were unaffected by impairment of JA defenses (*spr-2* and *def-1*) or constitutive activation of JA defenses (*35s::prosys*; **Figure 1**). The development of *B. tabaci* nymphs, however, was significantly accelerated by impairment of JA defenses and was substantially slowed by constitutive activation or artificial induction of JA defenses (**Figure 2**). These results are consistent with previous findings that impairment of JA defenses in *Arabidopsis* accelerates the development of *B. tabaci* nymphs (Zarate et al., 2007; Zhang et al., 2013b), and that activation of JA defenses in tomato slows nymphal development (Sánchez-Hernández et al., 2006). Our data also confirmed that JA defenses affect the development of *B. tabaci* nymphs under semi-field conditions (**Figure 6B**). Taken together, these results indicate that JA-regulated defenses are important in protecting tomato against *B. tabaci* nymphs but not adults.

It has been well-documented that the JA signaling pathway helps mediate plant defenses against herbivores in both laboratory and field (Ament et al., 2004; Kessler et al., 2004; Bosch et al., 2014). An appropriate JA-regulated defense response to herbivory depends on whether plants can recognize the elicitors or compounds released by herbivores during feeding or oviposition (Erb et al., 2012). Previous findings showed that whiteflies rarely puncture mesophyll cells, i.e., they avoid eliciting wound responses and contacting the potential defensive compounds that are stored within the vacuoles and apoplasts of these cells (Walling, 2000; Kempema et al., 2007). In the current study, however, feeding by *B. tabaci* adults induced the expression of JA-dependent genes (**Figures 4A–C**) and the accumulation of endogenous JA (**Figure 3A**) in the early of the infestation (6–48 h). Our data are partially consistent with previous findings that infestation by *B. tabaci* adults or application of their saliva activates the expression of the JA-dependent genes *LOX, AOS*, and *Chi9* in tomato at 24 h after infestation/application (Su et al., 2015). The induction of JA defenses by *B. tabaci* during initial feeding indicates that tomato plants recognize the elicitors derived from the insect’s oral secretions. Additional research is needed to identify *B. tabaci* elicitors and their mechanism(s) of action.

As the time of *B. tabaci* infestation increased in the current study, JA levels and the expression levels of two JA-dependent genes (*PI-I* and *PI-II*) declined to levels similar to those in non-infested leaves, and the expression level of another JA-dependent gene, *LoxD*, declined to levels that were even lower than those in non-infested leaves (**Figures 3A, 4**). This indicated that *B. tabaci* adults can suppress JA defenses after long periods of infestation. Regarding the underlying mechanisms, recent studies showed that the presence of the bacterial symbiont, *Hamiltonella defensa*, helps regulate the suppression of JA defenses by *B. tabaci* Mediterranean (MED), which is another cryptic species of *B. tabaci* (Su et al., 2015). Given that the MEAM1 species used in the current study also harbors *H. defensa* (Su et al., 2014), further experiments are needed to determine whether the suppression of JA defenses by the MEAM1 species of *B. tabaci* is mediated by *H. defensa* or other symbionts.

In contrast to the JA induction at initial period of adults feeding, *B. tabaci* nymphs did not activate the expression of JA-regulated genes (*LoxD, PI-I*, and *PI-II*) after 5, 7, or 10 days of infestation on tomato (**Figure 5**). This implied that *B. tabaci* nymphs induce different transcriptional responses than adults. Such transcriptional difference was also found in whitefly–squash interaction that *B. tabaci* nymph feeding induces the expression of S*LW1* and *SLW2*, whereas adult feeding does not (Van de Ven et al., 2000). In addition, the influence of insect developmental stage on plant responses to herbivores has been noted for volatile production in the maize–*Pseudaletia separata* interaction (Takabayashi et al., 1995).

Substantial evidence indicates that SA can reduce plant defenses against herbivory by interfering with steps in JA biosynthesis. In Lima bean, for example, SA blocked steps downstream of 12-oxo-phytodienoic acid (OPDA), an early intermediate of the JA-signaling cascade, and consequently suppressed the accumulation of endogenous JA (Engelberth et al., 2001). In *Arabidopsis*, SA inhibited the export of OPDA from the plastid to the cytosol and thereby prevented the further processing of OPDA to JA (Laudert and Weiler, 1998). In view of the induction of SA-dependent responses by whiteflies in many plant species (Zarate et al., 2007; Zhang et al., 2009; Rodríguez-Álvarez et al., 2015), it seems possible that the suppression of JA by whiteflies might be mediated in an SA-dependent manner, as proposed by Walling (2008). In the present study, the biosynthesis of JA was apparently not inhibited in *B. tabaci*-infested leaves, and JA content was higher in infested than in non-infested leaves during the first 48 h of infestation (**Figure 3A**). In the case of SA, however, the endogenous SA content during the first 48 h of infestation was too low to interfere with JA production (**Figure 3B**; Engelberth et al., 2001). The higher levels of SA that had accumulated at 72 h and thereafter apparently blocked the biosynthesis of JA in *B. tabaci*-infested leaves (**Figure 3B**). That JA suppression associated with SA induction was also observed in *B. tabaci*-infested leaves under semi-field conditions (**Figure 7A**). Moreover, if SA signaling was blocked, JA content increased in *B. tabaci*-infested leaves (**Figure 7B**), and nymphal development was delayed (**Figure 6A**). These data indicate the potential key role of SA in mediating the suppression of JA by *B. tabaci.* Our previous report indicated that the potential site of the JA and SA antagonism induced by *B. tabaci* might be located in the downstream of the JA signaling pathway (Zhang et al., 2013a). This speculation was also supported by recent findings that SA antagonizes JA signaling downstream of COI1, possibly by interfering with JA-regulated transcription factor ORA59, which was demonstrated to be degraded by SA (Van der Does et al., 2013).

In addition to suppressing JA defenses of tomato in the current study, recent findings show that *B. tabaci* adults prefer to oviposit on tomato plants that had been pre-infested with conspecifics (Su et al., 2018) or tomato plants in which JA signaling is blocked (Sánchez-Hernández et al., 2006). Such oviposition preference for infested tomato is highly related with the suppression of flavonoids by *B. tabaci* infestation (Su et al., 2018). Considering that flavonoids are known to have anti-herbivore function (Onkokesung et al., 2014), we believe that *B. tabaci* adults have acquired the ability to select the best food sources for their offspring. Given the high mobility of whiteflies in field, such host selection behavior of *B. tabaci* would facilitate its dispersion, establishment, and population growth in field.

## Author Contributions

P-JZ and X-PY designed the experiments. Y-CH and CZ conducted the experiments. P-JZ and Z-HY analyzed and interpreted the data. P-JZ and X-PY drafted and revised the paper. All authors read and approved the final manuscript.

## Conflict of Interest Statement

The authors declare that the research was conducted in the absence of any commercial or financial relationships that could be construed as a potential conflict of interest.
