# Supplementary material for: Jasmonic Acid-Dependent Defenses Play a Key Role in Defending Tomato Against Bemisia tabaci Nymphs, but Not Adults
Source: Front Plant Sci. 2018 Jul 20;9:1065. doi: 10.3389/fpls.2018.01065 (PMC6064940; doi:10.3389/fpls.2018.01065)
Supplement: Supplementary file 1 [file Table_1.DOC]

Table S1. Primers used for qRT-PCR.

| Gene | GenBank accession no. | Primer sequences | Reference |
| --- | --- | --- | --- |
| *LoxD* | U37840 | F: 5’-GCCCAAGTTTGCAGTGACAG-3’ | Heiz et al., 1997 |
|  |  | R: 5’-CTCGGGATCGTTCTCGTCAG-3’ |  |
| *PI-I* | K03290 | F: 5’-TGAAACTCTCATGGCACGAA-3’ | Kandoth et al., 2007 |
|  |  | R: 5’-TTTTGACATATTGTGGCTGCTT-3’ |  |
| *PI-II* | K03291 | F: 5’-CCCACGTTCAGAAGGAAGTC-3’ | Kandoth et al., 2007 |
|  |  | R: 5’-TTTTGGGCAATCCAGAAGAT-3’ |  |
| *PR-1b* | X14065 | F: 5’-CTCATATGAGACGTCGAGAAG-3’  R: 5’- GGAAACAAGAAGATGCAGTACTTAA-3’ | This study |
| *GAPDH* | U93208 | F: 5’-CTCCATCACAGCCACTCAGA-3’ | Milling et al., 2011， |
|  |  | R: 5’-TTCCACCTCTCCAATCCTTG-3’ |  |

References

Heitz, T., Bergey, D. R., Ryan, C. A. (1997). A gene encoding a chloroplast-targeted lipoxygenase in tomato leaves is transiently induced by wounding, systemin, and methyl jasmonate. *Plant Physiol.*114, 1085-1093

Kandoth, P. K., Ranf, S., Pancholi, S. S., Jayanty, S., Walla, M. D., Miller, W., Howe, G. A., Lincoln, D. E., Stratmann J. W. (2007). Tomato MAPKs LeMPK1, LeMPK2, and LeMPK3 function in the systemin-mediated defense response against herbivorous insects. *Proc Natl Acad Sci USA* 104, 12205-12210. DOI: 10.1073/pnas.0700344104

Milling, A., Babujee, L., Allen, C. (2011). *Ralstonia solanacearum* extracellular polysaccharide iIs a specific elicitor of defense responses in wilt-resistant tomato plants. *PLoS ONE* 6, e15853. DOI: 10.1371/journal.pone.0015853
