# Supplementary material for: Jasmonic Acid-Dependent Defenses Play a Key Role in Defending Tomato Against Bemisia tabaci Nymphs, but Not Adults
Source: Front Plant Sci. 2018 Jul 20;9:1065. doi: 10.3389/fpls.2018.01065 (PMC6064940; doi:10.3389/fpls.2018.01065)
Supplement: Supplementary file 2 [file Table_2.DOC]

Table S2. Number of nymphs survived on different tomato genotypes in the laboratory.

| Larval stage |  | Plant genotype | | | |  |  |
| --- | --- | --- | --- | --- | --- | --- | --- |
|  | CM (n=12) | spr-2 (n=12) | def-1 (n=15) | 35s::prosys (n=15) | CM (n=10) | JA (n=10) | SA (n=10) |
| 1^st^ instar | 7.4 ± 1.9 a | 5.1 ± 1.6 ab | 1.1 ± 0.6 b | 6.9 ± 2.0 a | 2.2 ± 0.8 b | 10.0 ± 1.6 a | 2.2 ± 0.6 b |
| 2^nd^ instar | 22.7 ± 2.5 a | 21.2 ± 2.2 a | 6.1 ± 1.1 b | 16.5 ± 2.3 a | 20.1 ± 1.4 a | 23.6 ± 2.6 a | 18.2 ± 1.9 a |
| 3^rd^ instar | 51.6 ± 2.9 a | 48.6 ± 3.1 a | 33.4 ± 2.1 b | 59.7 ± 3.0 a | 36.0 ± 1.9 a | 36.6 ± 1.8 a | 32.9 ± 1.8 a |
| 4^th^ instar | 30.6 ± 1.5 c | 41.1 ± 2.5 b | 66.1 ± 2.0 a | 15.5 ± 1.1 d | 36.7 ± 2.1 a | 26.8 ± 1.9 b | 39.3 ± 3.0 a |
| Total nymphs | 112.3 ± 3.4 a | 116.0 ± 3.3 a | 106.7 ± 2.9 a | 98.6 ± 3.7 a | 95.0 ± 4.9 a | 97.3 ± 6.1 a | 91.6 ± 6.4 a |

Values are means ± SE. Means followed by different letters are significantly different (*P* < 0.05; one-way ANOVA with Tukey’s multiple comparison).
