# Supplementary material for: Jasmonic Acid-Dependent Defenses Play a Key Role in Defending Tomato Against Bemisia tabaci Nymphs, but Not Adults
Source: Front Plant Sci. 2018 Jul 20;9:1065. doi: 10.3389/fpls.2018.01065 (PMC6064940; doi:10.3389/fpls.2018.01065)
Supplement: Supplementary file 3 [file Table_3.DOC]

Table S3. Number of nymphs survived on different tomato genotypes in semi-field.

| Larval stage |  | |  | | Plant genotype | | | |
| --- | --- | --- | --- | --- | --- | --- | --- | --- |
|  | CM (n=10) | spr-2 (n=10) | | def-1 (n=10) | | 35s::prosys (n=10) | MM (n=10) | NahG (n=10) |
| 1^st^ instar | 5.1 ± 0.9 b | 3.7 ± 0.6 b | | 1.3 ± 0.5 b | | 20.8 ± 1.1 a | 2.2 ± 0.6 b | 12.9 ± 1.3 a |
| 2^nd^ instar | 22.0 ± 1.9 b | 19.8 ± 1.9 b | | 18.3 ± 1.4 b | | 32.1 ± 1.2 a | 21.2 ± 1.5 b | 34.3 ± 0.9 a |
| 3^rd^ instar | 35.6 ± 1.3 a | 37.3 ± 2.1 a | | 38.2 ± 2.0 a | | 34.5 ± 1.9 a | 37.4 ± 1.5 a | 35.9 ± 1.5 a |
| 4^th^ instar | 19.6 ± 1.5 b | 24.2 ± 1.3 ab | | 28.9 ± 1.8 a | | 5.2 ± 0.84 c | 27.3 ± 1.0 a | 9.0 ± 1.2 b |
| Total nymphs | 82.3 ± 3.9 a | 85.0 ± 4.6 a | | 86.7 ± 3.9 a | | 92.6 ± 2.7 a | 88.1 ± 3.3 a | 92.1 ± 3.3 a |

Values are means ± SE. Means followed by different letters are significantly different (*P* < 0.05; one-way ANOVA with Tukey’s multiple comparison).
